# Advances in *Glycyrrhiza* polysaccharides: structure-activity relationship, immunomodulatory mechanisms, and therapeutic potential

**DOI:** 10.3389/fimmu.2026.1945961

**Published:** 2026-10-01

**Authors:** Xiaoling Ding, Wang Bo, Hongmei Gu, Xia Yang, Jie Yang, Hui Ma, Shan He, Xin Ma, Noun Dara, Xuebing Zhou

**Affiliations:** 1 People’s Hospital of Ningxia Hui Autonomous Region, Ningxia Medical University, Yinchuan, China; 2 Department of Pharmacy, General Hospital of Ningxia Medical University, Yinchuan, China

**Keywords:** *Glycyrrhiza* polysaccharides, gut microbiota, immunomodulation, structure-activity relationship, therapeutic potential

## Abstract

Immune dysregulation critically drives the pathogenesis of inflammation, malignancy, metabolic disorders, and autoimmunity, creating an urgent demand for safe, efficacious immunomodulatory interventions. *Glycyrrhiza* polysaccharides (GPs), bioactive macromolecules derived from licorice (*Glycyrrhiza* spp.), represent promising natural immunomodulators, although their structural determinants and mechanistic bases remain incompletely characterized. This review critical summarizes recent progress in GPs research regarding its structural characteristics, structure–activity relationships, immunomodulatory mechanisms, and translational potential. Relevant literature published between 1990 and 2026 was retrieved from multiple authoritative databases, including PubMed, Web of Science, ScienceDirect, Springer, and Google Scholar. We comprehensively outline how key structural properties including molecular weight, monosaccharide composition, glycosidic linkage patterns, branching architecture, chemical modification, and higher-order conformation are associated with, and may mechanistically contribute to GPs bioactivity, while strictly discriminating validated experimental findings from unconfirmed mechanistic hypotheses. We highlight that variable purity of conventional GPs preparations frequently confounds immunological results, emphasizing the necessity of rigorous endotoxin detection and proteinase digestion to verify polysaccharide-specific effects and exclude impurity-mediated interference. This review further dissects GP regulatory actions on innate and adaptive immune cells, including macrophages, dendritic cells, natural killer cells, T cells, and B cells, with particular attention to the microbiota–gut–immune axis. We also critically evaluate the preclinical potential of GPs as immunotherapeutic candidates, vaccine adjuvants, nutraceuticals, and agricultural additives, while explicitly distinguishing robust preclinical evidence from scarce clinical data. Current GPs immunomodulatory evidence is predominantly obtained from *in vitro* and animal models, with limited human studies and randomized controlled trials, restricting translational advancement. Collectively, this review highlights the importance of elucidating GPs structure–function principles, provides a refined framework for the standardized development, rational design and clinical translation of GPs-based biologics, and confirms that relevant evidence remains largely preclinical.

## 1 Introduction

The immune system is a highly coordinated network responsible for maintaining tissue homeostasis and defending organisms against infectious challenges, malignant transformation, and physiological stress (8, 12). However, persistent disruption of immune equilibrium contributes to the initiation and progression of a wide spectrum of diseases, including inflammatory diseases (102), cancer (62), metabolic disorders (69), and autoimmune conditions (7). Although immune-targeted therapies, such as cytokines, monoclonal antibodies, and immune checkpoint inhibitors, have revolutionized disease management, their broader application remains limited by immune-related toxicity, insufficient therapeutic durability, high production costs, and the complexity of achieving balanced immune regulation (29, 37, 81). These limitations have stimulated increasing interest in identifying naturally derived immunomodulatory molecules with improved safety profiles and multifunctional regulatory properties.

Natural polysaccharides derived from medicinal plants have emerged as important candidates in this context due to their excellent biocompatibility, structural diversity, and capacity to regulate immune responses through multiple biological interfaces (68, 101). Unlike single-target small molecules, polysaccharides can influence immune homeostasis by interacting with pattern recognition receptors (PRRs), regulating immune cell activation, and modulating the microbiota–gut–immune axis (85, 107). Nevertheless, the clinical translation of polysaccharide-based immunomodulators remains challenging because naturally occurring polysaccharides possess complex and heterogeneous structures, including variable molecular weight (MW), monosaccharide composition, glycosidic linkage patterns, branching characteristics, and three-dimensional conformations (40). Consequently, establishing precise structure–activity relationships (SARs) and determining how specific glycan structures govern immune recognition remain major unresolved questions in polysaccharide immunology (93).

*Glycyrrhiza* polysaccharides (GPs), isolated from licorice (*Glycyrrhiza* spp.), represent a particularly promising group of plant-derived immunomodulatory macromolecules. Licorice has been used extensively in traditional medicine and is recognized for its anti-inflammatory, hepatoprotective, and immune-regulatory properties (11). Previous pharmacological studies have primarily focused on low-molecular-weight compounds, especially glycyrrhizic acid and flavonoids; however, accumulating evidence indicates that GPs represent an important bioactive fraction contributing to the medicinal value of licorice (4). Recent investigations have revealed that GPs regulate diverse immune components, including macrophages, dendritic cells, natural killer cells, T lymphocytes, and B lymphocytes, while also exerting systemic immune effects through microbiota remodeling (1, 5). Furthermore, emerging studies have highlighted their potential applications as vaccine adjuvants, functional food ingredients, and sustainable agricultural immunomodulators (96).

Despite these advances, several critical scientific gaps continue to limit the development of GPs as precision immunomodulatory agents. First, although numerous GPs fractions have been isolated from different *Glycyrrhiza* species, the structural determinants responsible for variations in immunological activity remain insufficiently defined (99). Second, although GPs have been shown to activate immune cells through signaling pathways involving PRRs and inflammatory regulators, the molecular basis of GPs recognition and the relationship between structural motifs and receptor-mediated immune responses remain unclear (73). Third, accumulating evidence highlights the microbiota–gut–immune axis as a critical emerging mechanism underlying GP immunomodulation. Nevertheless, most current studies only establish correlational links among GPs intervention, microbial alteration, and immune modulation, while rigorous causal validation via germ-free models, antibiotic depletion, or fecal microbiota transplantation remains limited. Furthermore, how specific GP structural features shape microbial utilization, metabolite generation, and subsequent systemic immune regulation remains largely uncharacterized (121).

Addressing these knowledge gaps requires a comprehensive analysis that connects GPs structures with immunological mechanisms and translational applications. In this review, we summarize recent advances in the structural characterization of GPs, discuss emerging SARs, and examine their regulatory effects on immune cells and microbiota-mediated immune networks. We further evaluate the therapeutic potential of GPs, clearly distinguishing preclinical evidence from clinical evidence, and separate these data from the clinical literature on non-polysaccharide licorice constituents.

## 2 Structural characterization and structure–activity relationships

The immunomodulatory potential of GPs is intrinsically linked to their structural complexity, particularly MW, monosaccharide composition, glycosidic linkage patterns, higher-order conformation, and chemical modifications (113). Advances in analytical chemistry have enabled detailed characterization of these heteropolymers, yet the SARs of GPs remain incompletely resolved (38, 87). A major challenge is that structural features, extraction methods, and residual contaminants frequently co-vary, and impurities can independently trigger PRR signaling, leading to overestimation of polysaccharide bioactivity. Therefore, comparative studies of distinct GPs fractions often yield correlational trends rather than causal links. To rigorously evaluate the evidence, this review categorizes reported findings into the following categories. Overlapping structural and functional variations are described as ‘association’. Statistically validated relationships across comparable samples are described as correlation. Biologically plausible yet unconfirmed mechanisms are described as mechanistic hypotheses. Causal evidence is acknowledged only when supported by targeted structural manipulation, direct receptor binding, and functional perturbation assays. **Table 1** and **Figure 1** present the diverse chemical architectures of GPs, while **Figure 2** summarizes the current SARs framework under this grading system.

**Table 1:** Structural characterization of *Glycyrrhiza* polysaccharides.

GP fraction	Mw (kDa)	Monosaccharide composition	Structural feature	Analysis technique	Ref
GP	6.5	–	–	–	(75)
GP-1	7.6	Glc: Ara: Man: Gal = 98.03: 0.51: 0.75: 0.71	→4)-α-D-Glcp-(1→	HPGPC, PMP-HPLC, GC-MS, NMR, Congo red, SEM	(77)
LP-A	>100	–	–	Ethanol precipitation, Sevag deproteinization, dialysis, flow cytometry (FACS Calibur), Luminex 200 (cytokine bead panel)	(5)
GCP	55	–	–	HPGPC	(17)
GUPS-I	1.06	Rha: Xyl: Glc= 2.55:1.9:30.03	only α-glycosidic linkages	HPGPC, GC-MS, SEM, FT-IR, NMR	(3)
GUPS-II	29.1	Rha: Ara: Man: Glc: Gal = 1: 13.87: 1.59:16.76:15.72	α and β-glycosidic linkages	HPGPC, GC-MS, SEM, FT-IR, NMR	(3)
GUPS-III	14.9	Ara: Man: Glc: Gal = 1:5.33:1.91:5.97	α and β-glycosidic linkages	HPGPC, GC-MS, SEM, FT-IR, NMR	(3)
GPS-1	26.4	GalA: Ara: Rha: Gal: Man: Glc: Fuc: Xyl= 74.39: 10.12: 6.36: 4.85: 1.52: 1.34: 0.92: 0.51	HG-type pectin: backbone →4)-α-D-GalpA-(1→	HPGPC, HPAEC-PAD, GC-MS, NMR	(99)
GPs	Polydisperse, 3 peaks: Rt 8.25 min (4670 kDa); Rt 8.78 (2190 kDa); Rt 9.41 (871 kDa)	Rha: GalA: Glc: Gal: Ara = 1.00: 2.80: 31.8: 1.67: 3.73	–	HPGPC-ELSD, PMP-HPLC, FT-IR, SEM	(97)
GRPE-I	78.4–204.8	Gal: Ara: Rha: Glc: Man: Xyl: Fuc: GalA: GlcA= 40.1: 20.8: 9.2: 10.6: 4.7: 2.2: 1.5: 7.3: 3.7	→2)-α-L-Rha-(1→4)-α-D-GalA-	GPC, PMP-HPLC	(61)
GUP	–	Xyl: Man: Glc: Gal = 0.22: 1.20: 0.20: 1.00	–	UV, FT-IR, GC-MS, SEM-EDX, Congo red, XRD, TGA/DTG/DSC	(66)
GIP		Xyl: Man: Glc: Gal = 0.27: 0.97: 0.31: 1.00	–	UV, FT-IR, GC-MS, SEM-EDX, Congo red, XRD, TGA/DTG/DSC	(66)
GGP		Xyl: Man: Glc: Gal = 0.22: 1.22: 0.24: 1.00	–	UV, FT-IR, GC-MS, SEM-EDX, Congo red, XRD, TGA/DTG/DSC	(66)
GUPs-1	10.16	Glc: Gal: Ara= 23.4: 25.18: 8.32	–	HPGPC, GC-MS, FT-IR	(112)
GUPs-2	11.68	Glc: Gal: Ara: Rha: Man=14.0: 25.67: 17.54: 0.61: 0.68	–	HPGPC, GC-MS, FT-IR	(112)
GUPs-3	13.36	Glc: Gal: Ara: Rha: Man=1.13: 22.04: 31.44: 3.36: 1.95	–	HPGPC, GC-MS, FT-IR	(112)
GPN	38.7	Glc: Ara: Man: Gal = 98.03: 0.51: 0.75: 0.71	→4)-α-D-Glcp-(1→ and →4,6)-α-D-Glcp-(1→	HPLC, GC-MS, FT-IR, NMR, Congo red, SEM, XRD, TG/DTG/DSC	(54)
GCP-2	18.26	Man: Rha: GlcA: GalA: Glc: Gal: Ara= 2.10: 8.91: 5.27: 70.31: 3.90: 6.97:2.53	→4)-α-D-GalpA-(1→, →4)-GalpA-6-OMe-(1→	HPGPC, PMP-HPLC, GC-MS, FT-IR, NMR	(108)
GUP	–	Glc: GlcA: Gal = 0.902:0.056:0.043	contains t-Glcp, t-Galp, 1,2-Glcp, 1,4-Glcp, 1,6-Glcp, 1,6-Galp	FT-IR, GC-MS	(39)
GIBP	1960	Ara: Man: Glc: Gal = 1.00: 0.49: 4.05: 3.17	→4)-α-D-Glcp-(1→	HPGPC, PMP-HPLC, GC-MS, NMR	(59)
GP-LA	302.3	Fuc: Rha: Ara: Gal: Glc: Man: Xyl: GalA=0.69: 0.10: 8.71: 8.64: 71.7: 6.86: 10.52: 0.82	β-L-Araf-(1→, →5)-β-L-Araf-(1→, →4)-β-D-Xylp-(1→, β-D-Glcp-(1→, →3,4)-α-D-Glcp-(1→, →4,6)-α-D-Glcp-(1→	SEC-MALLS-VISC-RI, PMP-HPLC, GC-MS, FT-IR, NMR, Congo red, SEM, AFM, XRD	(33)
GP-HA	129.1	Fuc: Rha: Ara: Gal: Glc: Man: Xyl: GalA=0.65: 0.65: 5.55: 7.51: 78.38: 2.82: 3.96: 0.48	→6)-β-D-Galp-(1→, -α-D-Glcp-(1→, →3,4)-α-D-Glcp-(1→, →2,4)-α-D-Glcp-(1→, →4,6)-D-Glcp-(1→	SEC-MALLS-VISC-RI, PMP-HPLC, GC-MS, FT-IR, NMR, Congo red, SEM, AFM, XRD	(33)
AGP	2890	Rha: Ara: Xyl: Man: Glc: Gal = 1.00: 2.33: 2.85: 0.69: 3.05: 1.54	→6)-β-D-Glcp-(1→	HPLC, GC-MS, FT-IR, NMR, Congo red, CD, SEM, XRD	(114)
GPS-1-1	14.07	Glc: Ara: Gal = 91.42: 5.04: 3.55	→4)-α-D-Glcp-(1→	GC-MS, FT-IR, NMR	(56)

**Figure 1:**
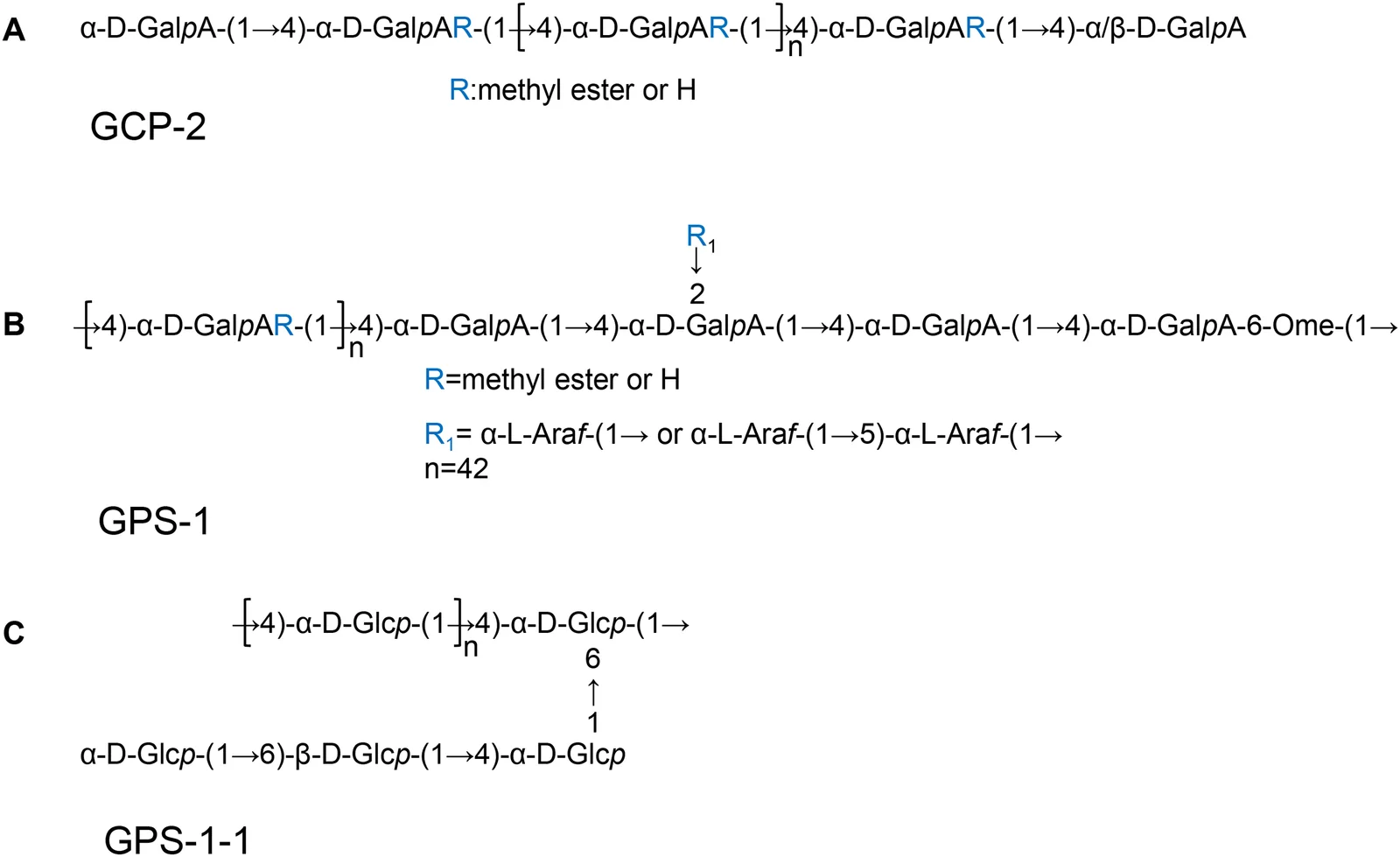
Representative structural features of polysaccharides from licorice. **(A)** The structural model of GCP-2. **(B)** The structural model of GPS-1. **(C)** The proposed structure of GPS-1-1.

**Figure 2:**
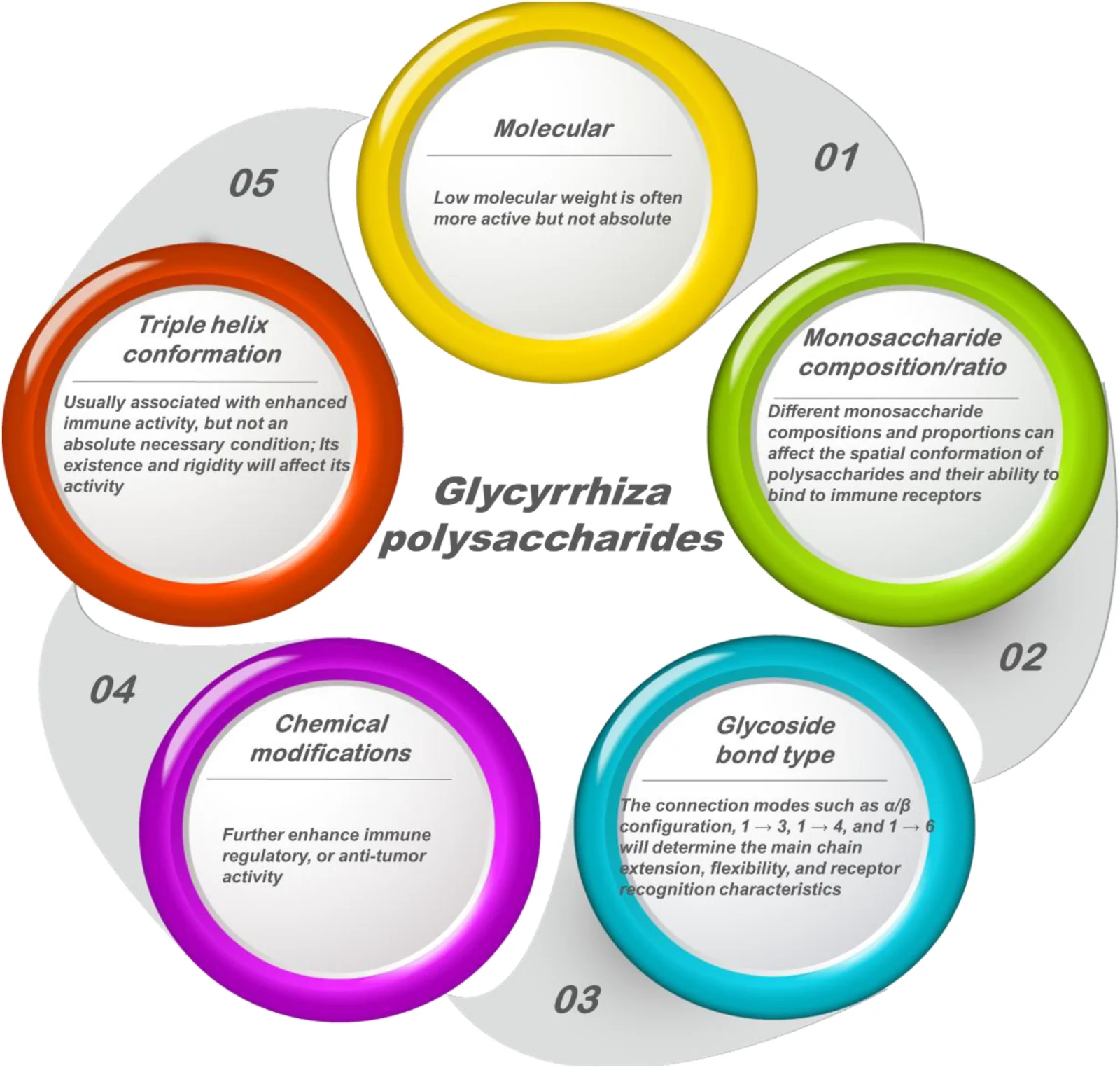
Structure activity relationship of immune regulatory activity of *Glycyrrhiza* polysaccharides.

### 2.1 Molecular weight affects immune regulation SARs

Molecular weight (MW) is a critical determinant of GPs immunomodulatory activity because it directly influences polysaccharide solubility, viscosity, spatial conformation, aggregation status, and cellular accessibility (34). However, accurate MW characterization faces technical challenges. Gel permeation chromatography (GPC), the standard method, relies on dextran calibration and only provides relative MW values. Structural discrepancies between irregular GPs conformations and linear dextrans introduce systematic errors, while the broad MW distribution of native GPs demands high-resolution fractionation. Consequently, reported MWs for GPs vary dramatically across studies, ranging from 5 kDa to 2890 kDa (114, 118, 120).

Despite this heterogeneity, several studies have attempted to correlate MW with immune function. Song et al. (75) verified the immunomodulatory capacity of a 6.5 kDa GPs fraction. In contrast, Aipire et al. (3) demonstrated that GUPS II (29.1 kDa) and GUPS III (14.9 kDa) induced stronger dendritic cell maturation and cytokine secretion than GUPS I (1.06 kDa). These findings suggest that intermediate MW fractions may exhibit superior immunostimulatory effects, but definitive conclusions are hindered by concurrent variations in monosaccharide composition, glycosidic linkages, and purity among the tested fractions. Future studies should employ well-matched GPs samples derived from identical parental fractions, selectively altering MW while controlling for other confounding variables to establish causal relationships.

### 2.2 Monosaccharide composition affects immune regulation SARs

Monosaccharide composition, as a core component of primary structure, fundamentally shapes the physicochemical traits and biological functions of GPs. GPs are heteropolysaccharides composed of neutral sugars, include glucose (Glc), galactose (Gal), arabinose (Ara), rhamnose (Rha), xylose (Xyl) and mannose (Man) and acidic residues (galacturonic acid (GalA) and glucuronic acid (GlcA)) in variable proportions (99, 118). This compositional diversity, influenced by botanical origin, processing methods, and extraction protocols, raises the question of whether specific monosaccharide profiles drive immunomodulatory activity. Emerging evidence indicates that both neutral and acidic sugars contribute to immune regulation. *Glycyrrhiza* UC, containing Ara, Glc, Gal, and Rha (10:30:27:1), enhanced reticuloendothelial system function (71). Sun et al. (77) isolated GP-1, a glucan that significantly restored immune function in immunosuppressed rat models and *in vitro* assays. Notably, acidic residues, particularly GalA, play a pivotal role. Wu et al. (99) investigated the immunomodulatory activity of GPS-1, a pectic polysaccharide rich in GalA (74.39%), together with Ara (10.12%), Rha (6.36%), Gal (4.85%), mannuronic acid (1.52%), Glc (1.34%), Fuc (0.92%), and Xyl (0.51%). The results demonstrated that GPS-1 activated dendritic cells through signaling pathways dependent on TLR2 and TLR4. This activation induced MAPK, NFκB, NOD, mTOR, and AMPK signaling cascades that promoted dendritic cell maturation and cytokine secretion. However, because endotoxin is a potent TLR4 agonist, these findings require endotoxin quantification and neutralization controls to confirm polysaccharide-specific activation. While the high GalA content of GPS-1 is compatible with a role for acidic pectic domains in DC activation, and motifs rich in Gal or Ara may engage PRRs, these remain mechanistic hypotheses until validated by direct binding experiments and gain-/loss-of-function studies using chemically or enzymatically edited, otherwise matched GPs fractions.

### 2.3 Chemical structures diversity affects immune regulation SARs

The anomeric configuration (α/β) and linkage positions (e.g., 1→3, 1→4, 1→6) critically determine chain flexibility, three-dimensional topology, and the ability to engage immune receptors (22, 32, 110). Modern characterization integrates classical degradative methods with advanced spectroscopic techniques, enabling the identification of several well-defined GPs fractions. Wu et al. (99) utilized NMR to identify multiple anomeric proton signals in GPS-1, indicative of diverse linkage configurations; they characterized GPS-1 as containing either an α-1,4-linked homogalacturonan (HG) domain or a rhamnogalacturonan-I (RG-I) region. Yu et al. (108) identified GCP-2 as an HG-dominated pectin with a core repeating unit of →4)-α-D-GalpA-(1→ and →4)-α-D-GalpA-6-OMe-(1→. In contrast, Niu et al. (56) described GPS-1-1, a neutral glucan featuring a backbone of →4)-α-D-Glcp-(1→ interspersed with branched →4,6)-α-D-Glcp-(1→ motifs. Similarly, Mutaillifu et al. (54) characterized GPN from *Glycyrrhiza* glabra, revealing a backbone of 1,4-linked Glc with complex branching. Collectively, these studies reveal that while GPs exhibit remarkable structural diversity, they share conserved architectural themes, predominantly constructed via α/β-(1→4) and/or (1→6) linkages, with side chains enriched in Ara, Rha, Gal, and GalA.

These specific glycosidic architectures directly correlate with immunomodulatory potency. Shimizu et al. (72) characterized GP-UA as possessing a β-(1→3)-D-glucopyranosyl backbone substituted with α-L-arabinose and β-(1→6)/β-(1→3)-linked D-galactose side chains. This specific architecture significantly enhanced murine peritoneal macrophage phagocytosis, indicating potent activation of the reticuloendothelial system; furthermore, GP-UA modulated complement activity, demonstrating that its glycosidic topology influences both cellular and humoral innate immunity. Similarly, Wu et al. (99) isolated GPS-1, a homogalacturonan-type pectic polysaccharide composed predominantly of (1→4)-linked α-D-galacturonic acid residues, which activated dendritic cells via TLR2 and TLR4 mediated signaling pathways, triggering multiple downstream immune cascades. Although evidence from other polysaccharide systems suggests that β-(1→3) or β-(1→6)-linked glucan motifs are immunologically active (64, 116), such findings should not be extrapolated as causal evidence for every GPs containing these linkages. Establishing definitive causal attribution requires matched linkage variants, selective enzymatic cleavage or derivatization, and direct assays for receptor binding and function.

### 2.4 Higher-order conformation and molecular morphology: characterization and SARs

Polysaccharide higher-order morphology and supramolecular architecture are increasingly acknowledged as critical determinants of biological functionality, characterizable via SEM, AFM, Congo red staining, and XRD (31, 41). Recent applications have elucidated the surface topography, aggregation behavior, and conformational dynamics of GPs. Using SEM, Rozi et al. (66) observed that GUP and GIP exhibited similar morphologies, consisting of smooth reticular frameworks formed by densely packed spherical granules, with the primary difference being particle size. In contrast, GGP displayed a smooth sheet-like morphology, attributed to differences in plant genotype and source tissue. Complementary Congo red assays demonstrated that all three seed-derived polysaccharides adopted a triple-helical conformation. While the co-occurrence of a triple-helical signal and antioxidant activity in these fractions is compatible with a contribution of higher-order conformation, genotype, tissue source, composition, and extraction history also differed.

Further insights emerge from studies isolating specific fractions. Guo et al. (33) extracted GP-HA and GP-LA, which shared a conserved →4)-β-D-Glcp-(1→ backbone but diverged in branching and conformation (both triple-helical). Morphologically, SEM revealed irregular spheroidal aggregates, while AFM distinguished a less-branched architecture for GP-HA from the interconnected network formed by GP-LA. XRD revealed that GP-LA was semi-crystalline, whereas GP-HA was largely amorphous. These features are associated with antioxidant and hypoglycemic readouts, highlighting the structural diversity of GPs. The triple-helix conformation, in particular, is a candidate contributor to immunomodulatory activity. Cheng et al. (20) observed that a semi-rigid preparation obtained after subcritical water treatment showed stronger macrophage-related activity than other treated preparations, suggesting an association between conformational state and activity. Likewise, the co-occurrence of a Congo red positive triple helix, immune readouts, microbiota changes, and SCFA changes in GP-1 is compatible with several mechanisms, but does not demonstrate direct receptor engagement by the triple helix (77). While the triple helix is beneficial in some plant polysaccharides (50), it is neither a universal requirement nor an independently validated determinant for GPs, as thermal processing concurrently alters MW, glycosidic integrity, and branching. Therefore, an optimal conformational stability window and direct or indirect triple helix mechanisms should be presented as hypotheses, pending causal testing via reversible conformational perturbation with preserved primary structure, followed by receptor binding and functional rescue experiments.

### 2.5 Structural modification and immunomodulatory activity

Native GPs face limitations including polydispersity and low solubility, necessitating structural modification to enhance therapeutic potential. Among these strategies, selenylation is particularly advantageous as it couples immune enhancement with improved selenium bioavailability. Direct comparisons indicate that selenylated polysaccharides demonstrate superior safety and bioactivity, producing stronger anti-inflammatory or immunological readouts than corresponding native preparations (89, 123). Beyond direct immune regulation, the enhanced antioxidant activity of Se-GUP may further contribute to immune homeostasis. Teng et al. (82) demonstrated that Se-GUP exhibited significantly stronger antioxidant activity than native GPs in both *in vitro* and *in vivo* models and effectively alleviated CCl_4_-induced acute liver injury. Because the liver is a central immunometabolic organ and oxidative stress is closely associated with immune dysfunction, this hepatoprotection may indirectly support immune function by preserving hepatic function and reducing oxidative damage. However, it remains unclear which feature related to modification caused the difference. Thus, selenylation should be described as a promising strategy for perturbing structure, rather than proof that selenium incorporation per se is the active determinant. Future research should explore other modifications and prioritize deciphering how specific chemical alterations translate into functional outcomes using matched controls and detailed structural reporting.

Collectively, the structural features of GPs, spanning molecular weight, monosaccharide composition, glycosidic linkage patterns, higher order conformation, and chemical modification, are consistently associated with immunomodulatory readouts, yet definitive causal relationships remain scarce. The principal obstacles to definitive SARs are as follows. First, multiple structural features covary across separately isolated fractions. Second, variable purity and residual contaminants can independently trigger PRR signaling. Third, direct receptor binding assays are nearly absent. Fourth, there is a lack of matched preparations with selectively varied single structural parameters. Overcoming these obstacles will require homogeneous GPs standards, systematic impurity controls, and integrated approaches that combine structural manipulation, receptor binding, and functional perturbation. **Figure 2** summarizes the current understanding of relationships between specific structural features and their corresponding immunomodulatory mechanisms under the established evidence grading framework.

## 3 Immunological activity

Immunomodulation represents the defining bioactivity of GPs. Although GPs exert pleiotropic effects throughout the innate and adaptive immune systems (**Table 2**), current data suggest their principal mode of action is macrophage-centric. Macrophage activation subsequently bridges innate and adaptive immunity through robust cytokine production and antigen presentation.

**Table 2:** Immune regulatory activity of *Glycyrrhiza* polysaccharides.

Polysaccharides.	Types	Experimental model	Dosage	Administration route	Treatment duration	Toxicity assessment	Physiological/translational relevance	Effect (increase, ↑; decrease, ↓)	Ref
LMW-GP	*In vivo*	BALB/c mice,	GPL: 100 mg/kg bw; GPH: 200 mg/kg bw	Oral (po)	14 days	No overt adverse effects described	Doses correspond to ~8–16 mg/kg human equivalent dose (HED), showing moderate translational relevance for intestinal immune modulation	Body weight ↑, food intake ↑, colon length ↑, villus length ↑; goblet cells ↑, mucin ↑; colon CD4^+^ T ↑, CD8^+^ T ↑; serum IL-2, IL-10, TGF-β3, IgM, IgG ↑; colon sIgA ↑; colon p38↓, ERK↓, NF-κBp50↓; Proteobacteria ↓; SCFA total ↑	(75)
LP	*In vivo*	CT-26 colon carcinoma–bearing BALB/c mice	500 mg/kg bw/day, po, 14 d (CTX positive: 50 mg/kg ip, 10 d)	Oral (po)	14 days (CTX control: 50 mg/kg ip, 10 d)	Not reported; no overt toxicity at tested dose	500 mg/kg mouse dose ≈ 40.6 mg/kg HED; high-dose setting limits direct dietary translation	Body weight (no-tumor) ↑; Tumor weight ↓; Spleen ↑, Thymus ↑; Thymus index ↑; CD4^+^ ↑, CD8^+^ ↓; Serum IL-2 ↑, IL-6 ↑, IL-7 ↑ (vs Saline), TNFα ↓	(5)
GPS-1	*In vivo*	Hy-Line brown chickens	GPS-L: 300 mg/kg bw/day, po; GPS-M: 450 mg/kg; GPS-H: 600 mg/kg; 14 d	Oral (po)	14 days	Not reported; no overt adverse effects described	Veterinary-relevant dose range; applicability to mammals requires further validation	IL-2 ↑, IL-4 ↑, IFN-γ ↑, IL-17 ↑, CD4^+^/CD8^+^ expression ↑	(5)
GPS	*In vivo*	Hy-Line Brown chickens	GPS-L: 300 mg/kg bw/day, po; GPS-M: 450 mg/kg; GPS-H: 600 mg/kg; 14 d	Oral (po)	14 days	Not reported; no overt adverse effects described	Veterinary-relevant dose range; mammalian translation not established	IL-2 ↑, IL-4 ↑, IL-1β ↑, IFN-γ ↑, *Firmicutes* ↓, *Bacteroidetes* ↑, F/B ↓, *Actinobacteria* ↑	(98)
NGUP	*In vitro* and *In vivo*	*In vitro*: C57BL/6 mice, splenic T co-culture; *In vivo*: C57BL/6 mice	*In vitro*: 0.5/5/50 μg/mL; *In vivo*: NGUPL (low)@OVA: NGUP 50 + OVA 20 μg/dose; NGUPL (high)@OVA: NGUP 200 + OVA 20 μg/dose; AL+OVA: Al 25 μg + OVA 20 μg; 3-dose id	*In vitro: cell culture; In vivo: 3-dose immunization*	*In vitro*: 36 h; *In vivo*: 3-dose prime-boost schedule	Not reported	*In vitro* concentrations at 0.5–5 μg/mL are physiologically plausible; 50 μg/mL is supraphysiological. *In vivo* adjuvant doses show preliminary translational relevance	*In vitro* BMDC: CD80/CD86/MHCII ↑; IL-12p70, TNF-α, IL-6 ↑; SIINFEKL-H2Kb ↑; TLR4/MyD88/TRAF6 mRNA ↑, NF-κB p65 phosphorylation ↑; *In vivo*: DC ↑, CD69^+^CD4^+^/CD8^+^ ↑, IFN-γ^+^CD8^+^ ↑, CD4^+^ Tmem ↑, CD8^+^ Tmem ↑	(105)
GUPS	*In vitro* and *In vivo*	*In vitro*: human monocyte-derived DCs, murine BM-DCs; *In vivo* naive: BALB/c mice	*In vitro*: 1, 5, 10, 20, 50 μg/mL; naive: 100 μg/ip single; CTX model: 20 mg/kg ip ×7 d	*In vitro*: cell culture; *In vivo*: intraperitoneal (ip)	*In vitro*: 24–48 h; *In vivo*: 7 days (tumor model)	Not reported	*In vitro* 1–10 μg/mL range is physiologically relevant; 50 μg/mL is supraphysiological. IP route bypasses GI absorption, limiting oral translation	CD86/HLA-DR ↑; CD40, CD80, CD86, MHC-I/II/CCR7 ↑; IL-1β, IL-12p40, TNF-α ↑; FITC-dextran endocytosis ↓; p-ERK, p-JNK, p-p38, p-IKKα/β, p-IκBa, p-NF-κBp65 ↑	(2)
GCP	*In vivo*	BALB/c mice	500 mg/kg bw/day	Oral (po)	14 days	Not reported; no overt toxicity described	500 mg/kg mouse dose ≈ 40.6 mg/kg HED; high-dose pharmacological effect	Tumor: tumor weight ↓, Ki67^+^ ↓; Systemic: spleen index ↑; splenic CD3^+^ ↑, tumor-infiltrating CD3^+^ ↑; Intestinal γδT (flow): CD3^+^γδT ↑, NKG2D(high) γδT ↑, CD27(high) γδT ↑, intracellular IFN-γ^+^ γδT ↑; splenic γδT (IHC) ↑, tumor-infiltrating CD27^+^ γδT ↑; Serum cytokines: IL-7 ↑, IFN-γ ↑, IL-12 ↑, TNF-α ↑; Gut mucosal cytokines: IL-2 ↑, IL-7 ↑, IL-12 ↑, IL-21 ↑, TNF-α ↑; phyla: *Bacteroidetes* ↓, *Firmicutes* ↑, *Proteobacteria* ↑, *Tenericutes* ↓, *Verrucomicrobia* ↑, *Epsilonbacteraeota* ↓ vs Model; genus: *Bacteroides*, *Alloprevotella*, *Lachnospiraceae*_*NK4A13*6↓, *Lactobacillusrecovered*, *Parabacteroides*↑; TLRs/NF-κB (gut mucosa): TLR2, TLR4, TLR6 mRNA ↑; p-IκBα protein ↑, NF-κB p65 ↑	(17)
GPS-1	*In vitro* and *In vivo*	*In vivo*: ICR mice; *In vitro*: mouse BM-DC	*In vivo*: 300/450/600 mg/kg/d po ×14 d; *In vitro*: 125/250/500 μg/mL, 36 h	*In vitro: cell culture; In vivo: Oral (po)*	*In vitro*: 36 h; *In vivo*: 14 days	Not reported	*In vitro* concentrations are supraphysiological. *In vivo* doses (300–600 mg/kg) ≈ 24–49 mg/kg HED, pharmacological dose range	*In vivo* serum: IFN-γ↑, IL-10↑ dose-dep; histology: duodenum/jejunum/ileum villi↑(GPS-H strongest), spleen germinal center↑(GPS-M/H), thymic cortex↑(GPS-M/H); spleen T: CD3^+^CD4^+^ 4.09→5.13%(+25.43%), CD3^+^CD8^+^ 2.76→3.92%(+42.03%); *In vitro* DC: CD80/CD86↑; IFN-γ, TNF-α, IL-1β, IL-10↑ dose-dep; OVA-FITC phagocytosis↑	(99)
GPs	*In vivo*	Wistar rats	GP-L: 0.52 g/kg/d; GP-H: 2.10 g/kg/d; POS: 40 mg/kg/d; CTX 60 mg/kg ip ×3 d	Oral (po)	14 days	Not reported; no overt adverse effects described	High doses (0.52–2.10 g/kg) correspond to ~42–170 mg/kg HED; supraphysiological, proof-of-concept only	Body/organ: BW↑; spleen index↑; thymus index↑; Cytokines (serum): IFN-γ↑, IL-6↑, IgG↑, IL-10↑, IL-2↑; Intestinal barrier (ileum IF): ZO-1↑, occludin↑; Genus: *Allobaculum*, *Dubosiella*, *norank:o_Clostridia_UCG-014*, *g:norank_o:_RF39*↑, *Enterococcus*↓; SCFA (fecal): propionate, iso-butyrate, 2-methyl-butyrate, iso-valerate ↑; SCFA (serum): propionate, iso-butyrate, butyrate, 2-methyl-butyrate ↑	(97)
LP Fractions A/B/C	*In vitro*	Mouse intestinal epithelial cells (IEC-6) and mouse colon carcinoma (CT-26)	IEC-6/CT-26: 0.01–0.1 mg/ml screened; ED_50_ IEC-6 ≈ 100 μg/ml; CT-26 inhibition at ≤50 μg/ml; IL-7 qPCR: 100 μg/ml; T-lymph: supernatant from 100 μg/ml-treated IEC-6, 6 h	Cell culture	24–48 h	Not reported	Concentrations ≥50 μg/mL are supraphysiological for systemic exposure; may be relevant for luminal intestinal exposure	IEC-6: proliferation ↑; CT-26: proliferation ↓: IL-7: IEC-6 mRNA ↑; T lymphocyte: supernatant ↑	(6)
GPs	*In vivo*	SPF Kunming mice	GPL: 100; GPM: 200; GPH: 400 mg/kg/d po ×14 d; APS: 200 mg/kg/d po	Oral (po)	14 days	Not reported; no overt toxicity described	100–400 mg/kg mouse ≈ 8–32 mg/kg HED; moderate translational relevance for antiviral immune support	Serum cytokines: TNF-α↓, IL-6 ↓, IL-4 ↑, IL-10 ↑; Barrier: serum D-lactate ↓, DAO ↓, intestinal sIgA ↑; ZO-1/Occludin mRNA↑, protein (IF/WB) ↑; Viral load: PRV gE qPCR ↓; Microbiota: α-diversity ↑; *Lactobacillus*, *Bacteroides*↑; *Prevotella*↓	(74)
GPN	*In vitro* and *In vivo*	*In vitro*: Caco-2 cells; *In vivo*: ICR mice	*In vitro* DC: 50 μg/mL; *In vivo*: 100 mg/kg/d po ×7 d	*In vitro*: cell culture; *In vivo*: Oral (po)	*In vitro*: 24–48 h; *In vivo*: 7 days	Not reported	*In vitro* 50 μg/mL is supraphysiological. *In vivo* 100 mg/kg ≈ 8.1 mg/kg HED, more physiologically relevant	BM-DC: CD86↑, CD40↑; IL-12p40↑, TNF-α↑; T, B, NK cells↑	(100)
GRPE-I	*In vitro* and *In vivo*	*In vitro*: murine peritoneal macrophage; *In vivo*: BALB/c mice	*In vitro*: 1, 10, 100 μg/mL, 24 h; *In vivo*: 12.5, 25, 50 mg/kg/d po ×15 d	*In vitro: cell culture; In vivo: Oral (po)*	*In vitro*: 24 h; *In vivo*: 15 days	Not reported	*In vitro* 1–10 μg/mL is physiologically plausible; 100 μg/mL is supraphysiological. *In vivo* doses show moderate translational relevance	*In vivo* clinical: BW loss attenuated dose-dep; Thymus: NC↓; Peritoneal macro: proliferation ↓; phagocytosis ↑ dose-dep; NO↑, IL-6↑; Serum: IgA↑, IgG↑ dose-dep, IL-6↑, TNF-α↑, IFN-γ↑; Spleen tissue: IgA/IgG trend↑, IL-6/TNF-α/IFN-γ ↑ consistent with serum; WB(spleen): p-ERK↓, p-p38↓, IκBa↓; SCFA (fecal + cecum): acetate, propionate, butyrate ↓	(61)
GCP	*In vivo*	BALB/c mice	500 mg/kg/d po ×14 d	Oral (po)	14 days	Not reported	500 mg/kg ≈ 40.6 mg/kg HED; pharmacological high-dose effect	tumor weight ↓, spleen index ↑, *Enterorhabdus*↑, *Odoribacter*↑, *Ruminococcaceae*_UCG_014↑, *Ruminococcaceae*_UCG_010↑, *Enterococcus*↑, *Ruminiclostridium*_5↑, *Parasutterella*↓, *Clostridium*_*sensu*_*stricto*_1↓, *Blautia*↓	(115)
GP-1	*In vivo* and *In vitro*	*In vivo*: Wistar rats; *In vitro*: rat fecal fermentation	*In vivo*: 0.84, 3.36 g/kg/d po ×14 d; *In vitro*: 2, 10 mg/mL, 12 h	*In vitro: fecal culture; In vivo: Oral (po)*	*In vitro*: 12 h; *In vivo*: 14 days	Not reported	Very high *in vivo* doses (0.84–3.36 g/kg) ≈ 68–273 mg/kg HED; supraphysiological, proof-of-concept only	*In vivo*: IL-2↓trend, IL-6↓trend, TNF-α↓, IL-4↑, IgA↑, IgG↑; *In vitro*: *genus Lactobacillus*↑, *Escherichia*-*Shigella*↓, *Dubosiella*↑; SCFA: acetate↑, propionate↑, isovalerate↑	(77)
GP1	*In vitro* and *In vivo*	*In vitro*: RAW 264.7 cell; *In vivo*: BALB/c mice	*In vitro*: 5, 10, 25, 50, 100, 200, 500, 1000, 2000, 5000 μg/mL; *In vivo*: 200, 800 mg/kg/d po ×8 wk	*In vitro: cell culture; In vivo: Oral (po)*	*In vitro*: 24–48 h; *In vivo*: 8 weeks	Not reported	*In vitro* concentrations ≥25 μg/mL are supraphysiological. *In vivo* 200 mg/kg ≈ 16.3 mg/kg HED, moderate relevance	*In vitro*: Macrophage phagocytosis ↑, IL-1β, IL-6, TNF-α ↑; *In vivo*: liver index ↑, spleen index ↑, splenic T Lymph ↑, splenic macro ↑, colonic T ↑, IFN-γ ↑, TGF-β3 ↑, IgG ↑, IgM ↑, IL-1β ↓, IL-2 ↓, IL-4 ↓, IL-10 ↓, TNF-α ↓, MCP-1↓, MIP-1α↓, *Chao1* ↑, *Shannon* ↑; *Bacteroides* ↑, *Firmicutes* ↓, *Proteobacteria* ↓; genus: *Muribaculaceae_unclassified* ↑, *Muribaculum* ↑, *Prevotellaceae_UCG-001* ↑, *Paramuribaculum*↑, *Muribaculum_sp_H5* ↑	(76)

All entries in this table belong to *in vitro*, ex *vivo*, or animal-study categories. Human equivalent dose (HED) estimates are based on standard FDA body surface area conversion (mouse: ÷12.3; rat: ÷6.2) and are provided for reference only.

### 3.1 Vaccine adjuvant

As frontline effector cells of innate immunity, macrophages bridge innate and adaptive immune responses through their functional plasticity, characterized by dynamic polarization into classically activated (M1) or alternatively activated (M2) phenotypes (10, 51). Consequently, macrophages have become one of the most extensively studied target cells for investigating the immunomodulatory activity of GPs. Accumulating evidence, predominantly from *in vitro* studies and animal models, demonstrates that GPs potentiate macrophage activation and effector functions. Nitric oxide (NO) production serves as a key proxy for macrophage activation; early work by (57) established that crude polysaccharides from *G. glabra* and *G. uralensis* induce dose-dependent NO synthesis in murine macrophages. Subsequent studies confirmed these findings in primary peritoneal macrophages both *in vitro* and *in vivo*, while polymyxin B treatment ruled out endotoxin contamination, attributing the immunostimulatory effects directly to GPs (18). Beyond activation, GPs significantly enhance phagocytic clearance, as evidenced by increased neutral red uptake and elevated secretion of pro-inflammatory cytokines (IL-1, IL-6, IL-12, and TNF-α) (19, 48). Furthermore, Cong et al. (24) further noted rapid upregulation of TNF-α and IL-1β within 24 hours of stimulation, indicating swift initiation of innate defenses. Mechanistic studies have demonstrated that these immunomodulatory effects are predominantly mediated via TLR4-dependent signaling pathways. GPs-triggered TLR4 activation, which is verified by TLR4 blockade and gene silencing assays rather than direct receptor-binding experiments, initiates both MyD88-dependent and MyD88-independent signaling cascades and ultimately induces NF-κB activation (88, 92). For example, Cong et al. (25) demonstrated that GiP-B1 from G. inflata activates RAW264.7 cells via the TLR4/MyD88/NF-κB axis; genetic silencing of TLR4 abrogated this response, supporting a non-redundant role for TLR4 in this signaling pathway. Notably, these data establish TLR4 pathway involvement but do not demonstrate direct GPs–TLR4 ligand-receptor binding, which would require dedicated binding assays However, because LPS is also a classic TLR4 agonist, TLR4-dependent activation alone cannot distinguish GPs-specific effects from endotoxin-mediated effects unless endotoxin levels are quantified and neutralization controls are performed. Although Cheng et al. used polymyxin B to rule out endotoxin contamination in their preparation, not all subsequent studies reporting TLR4-dependent macrophage activation have included equivalent controls, and this limitation should be borne in mind when interpreting the mechanistic data (18).

Intriguingly, GPs-mediated effects are context-dependent rather than uniformly pro-inflammatory. Under pathological conditions, GPs can suppress aberrant macrophage activation to restore homeostasis. Li et al. (45) demonstrated that in a co-culture model mimicking the tumor microenvironment, GPs inhibited STAT3 phosphorylation in macrophages. Given that persistent STAT3 activation drives immunosuppressive M2-polarized tumor-associated macrophages (TAMs), this inhibition suggests GPs can remodel the tumor immune microenvironment. Thus, in all *in vitro* and co-culture experimental models investigated so far, GPs exert dual immunomodulatory effects. Specifically, they enhance innate immune responses under physiological basal conditions and alleviate pathological immunosuppression within disease-specific microenvironments. Whether such dual regulatory properties can be recapitulated in human systems requires further validation.

### 3.2 Dendritic cells

Dendritic cells (DCs), the preeminent professional antigen-presenting cells, serve as the critical bridge between innate and adaptive immunity by orchestrating T-cell priming and polarization (90). Consequently, modulation of DC ontogeny and maturation represents a principal mechanism underlying GPs-driven adaptive immune responses. Current evidence indicates that GPs regulate multiple facets of DC biology, spanning monocyte differentiation, phenotypic maturation, and functional activation. Tang et al. (80) demonstrated that GPs promote the differentiation of human monocytes into DCs under defined cytokine conditions, suggesting a role in early myeloid lineage specification. Similarly, Zhang et al. (117) reported that GPs dose-dependently induced the differentiation of umbilical cord blood mononuclear cells into mature DCs, characterized morphologically by enlarged cell bodies, cytoplasmic spreading, and abundant dendritic projections effects peaking at 400 μg/mL. Phenotypically, GPs-treated DCs exhibit upregulated expression of MHC-II, CD80, CD86, and CD40 (124). These molecules provide the co-stimulatory signals required for productive DC–T cell interactions, and their increased expression reflects an enhanced capacity of DCs to initiate adaptive immune responses. The molecular mechanisms underlying GPs-induced DC maturation are primarily mediated through PRRs-dependent signaling pathways. Current evidence implicates TLR4 as the principal receptor associated with GP recognition, although direct GPs–TLR4 binding studies remain limited and endotoxin controls are essential for definitive attribution. TLR4 activation further initiates downstream MAPK and NF-κB signaling cascades, which ultimately promote DC maturation. Aipire et al. (2) substantiated this by showing that GPs activate the TLR4/p38/JNK/NF-κB signaling axis, which is indispensable for DC functional activation; as with other GPs–TLR4 studies, these data demonstrate pathway dependence rather than direct ligand-receptor binding. Consistent with findings from macrophage-related research, the interpretation of PRR-dependent DC maturation, especially TLR4-driven cellular activation requires rigorous caution. Reliable conclusions cannot be drawn without accurate endotoxin quantification and the inclusion of polymyxin B treatment or validated endotoxin-neutralizing controls. Furthermore, residual proteins and glycoproteins present in GPs extracts may trigger DC activation via alternative PRR signaling pathways, further highlighting the critical necessity of comprehensive purity assessment for all relevant experimental studies. Functionally, GPs markedly enhance the cytokine-producing capacity of DCs, thereby strengthening innate-adaptive crosstalk. Notably, IL-12 production is significantly elevated in a dose-dependent manner following GPs treatment, with high-dose regimens nearly doubling secretion compared to low-dose controls (3, 117) This robust IL-12 induction, observed both *in vitro* and *in vivo*, underscores its role in promoting Th1 polarization and cytotoxic T lymphocyte (CTL) priming. Recent studies further elucidate this axis, Nadiremu et al. (55) demonstrated that purified GiP-B1 from *G. inflata* promotes NF-κB p65 phosphorylation and IL-12 expression, thereby activating the IL-12/NF-κB/STAT4 signaling cascade to drive Th1-oriented immunity.

Collectively, current experimental evidence, which is predominantly obtained from *in vitro* and ex *vivo* assays utilizing human monocyte-derived and cord blood-derived DCs, with only limited validation in murine *in vivo* models, demonstrates that GPs modulate multiple biological functions of DCs, including cellular differentiation, phenotypic maturation, cytokine production, and subsequent T cell polarization. Nevertheless, current mechanistic studies have largely focused on TLR4 signaling, whereas the potential contributions of other PRRs, receptor cooperation, and GPs structural heterogeneity remain poorly understood. Future studies integrating precise structural characterization with single-cell immunology and receptor-binding analyses will be instrumental in defining how specific GPs structural motifs regulate DC function and adaptive immune responses.

### 3.3 Natural killer cells

Natural killer (NK) cells are effector lymphocytes of the innate immune system that mediate rapid, antigen-independent surveillance against virally infected and transformed cells (21). Enhancing NK cell cytotoxicity is therefore a key mechanism underlying the immunomodulatory and antitumor effects of GPs.

Accumulating evidence demonstrates that GPs significantly enhance NK cell activity in both normal and immunocompromised animal models. For example, Chen et al. (16) reported that *Glycyrrhiza uralensis* polysaccharides improved overall health status *in vivo*, as evidenced by increased body weight and serum IL-2 levels. Crucially, these polysaccharides also restored the CD4^+^/CD8^+^ T-cell ratio and markedly enhanced NK cell cytotoxic activity in these animal models, underscoring their preclinical potential as antitumor immunomodulators. Unlike macrophages or dendritic cells, NK cells are not directly activated by antigen recognition but are highly responsive to cytokine cues. Consequently, GPs enhance NK cell function primarily by orchestrating a permissive cytokine landscape. Wu et al. (99) demonstrated that GPs stimulate the production of key immunoregulatory cytokines, notably IFN-γ—the signature effector cytokine of NK cells essential for antiviral and antitumor immunity. Furthermore, GPs drive a systemic shift toward a Th1-dominant environment by upregulating IL-2 and IL-12 while concomitantly suppressing the immunosuppressive cytokines IL-10 and TGF-β (5, 36). Since IL-2 and IL-12 are critical for NK cell proliferation and IFN-γ production, whereas IL-10 and TGF-β are inhibitory, this cytokine reprogramming creates a milieu highly conducive to NK cell cytotoxicity. This indirect immunomodulatory axis is further supported by observations that GPs regulate IL-6 and IL-7 production (83). Given that IL-7 is indispensable for NK cell development, survival, and long-term functional maintenance (23), this suggests GPs support NK cell fitness at multiple levels. Moreover, by promoting anti-inflammatory cytokines while tempering excessive pro-inflammatory responses, GPs maintain immune homeostasis, providing a balanced environment for sustained NK cell activity (1, 73).

In summary, current evidence suggests that GPs enhance NK cell-mediated immunity predominantly through indirect mechanisms involving cytokine network remodeling, rather than via direct receptor-ligand interactions. However, the molecular intricacies of GPs–NK cell crosstalk remain poorly defined. Specifically, whether GPs can directly engage specific NK cell surface receptors or act solely through accessory cells warrants further investigation. Future studies integrating single-cell receptor profiling, co-culture models, and structure–activity relationship analyses are essential to delineate the precise mechanisms by which GPs potentiate NK cell surveillance.

### 3.4 T lymphocyte subsets

T cells are central to adaptive immunity, and their dysregulation contributes to numerous pathologies. GPs modulate T-cell responses, offering therapeutic potential. Early studies primarily focused on the effects of GPs on global T-cell activation and proliferation. Remela and colleagues (65) demonstrated that GPs potentiate the proliferative response of concanavalin A-stimulated T lymphocytes, providing seminal evidence for GPs-mediated modulation of adaptive immunity. Similarly, Wu et al. (99) reported that the purified fraction GPS-1 expanded both CD3^+^CD4^+^ helper and CD3^+^CD8^+^ cytotoxic T-cell populations in the spleen while promoting IFN-γ and IL-4 production. These findings suggest GPs coordinately regulate T-cell expansion, functional differentiation, and cytokine polarization. Subsequent studies delineated subset-specific effects. Sun et al. (78) showed that GPs enhance the proliferation, cytokine secretion, and cytotoxic potential of human peripheral blood γδ T cells, highlighting their capacity to boost innate-like T-cell-mediated antitumor immunity. In a complementary *in vivo* study, Ayeka et al. (5) demonstrated that GPs administration in CT26 tumor-bearing mice bolstered both CD4^+^ and CD8^+^ T-cell responses, concomitant with significant tumor growth inhibition and survival prolongation. Together, these data, obtained from *in vitro* lymphocyte assays and CT26 tumor-bearing mouse models, indicate that GPs enhance antitumor immunity by synergistically promoting helper and cytotoxic T-cell effector functions; confirmation in human studies is still lacking.

Recent studies have further expanded the mechanistic framework of GPs-mediated T-cell regulation by incorporating the gut microbiota as an important intermediary. In a rigorous causal validation study, Song et al. (75) reported that low-molecular-weight GPs altered the gut microbiota and were associated with changes in systemic CD4^+^ and CD8^+^ T-cell responses. Because microbiota transfer into germ-free recipients reproduced selected immune phenotypes, the study supports a microbiota-dependent component of GPs-mediated T-cell regulation in this experimental model. However, the transfer experiment does not establish that all observed T-cell effects are microbiota-mediated, and the responsible microbial taxa or metabolites remain to be defined. Consistent with this concept, Tin et al. (83) observed that GPs treatment increased the cellularity of splenic immune compartments, including CD3^+^ T cells, CD11c^+^ dendritic cells, CD11b^+^ myeloid cells, and CD19^+^ B cells, with a particularly notable expansion of activated CD44^+^CD8^+^ effector T cells. Mechanistically, Chen et al. demonstrated that GPs exert dual antitumor effects by directly triggering the TLR/NF-κB cascade in γδ T cells and remodeling gut microbiota to sustain γδ T cell activation (17). However, such TLR activation requires careful interpretation, as residual LPS impurities in GPs preparations can independently activate this pathway and cause false-positive immune responses. Furthermore, Han et al. (35) demonstrated that phosphorylated GPs exhibit enhanced immunomodulatory activity compared to native GPs in avian models, providing proof-of-concept that rational structural modification can optimize T-cell regulatory capacity. Collectively, current preclinical evidence demonstrates that GPs act as multifunctional modulators of T-cell immunity. Future structure–activity relationship studies should clarify how specific glycan structural traits govern T-cell subset specificity, with rigorous stratification of correlational observations, mechanistic hypotheses, and experimentally validated causal relationships. Integrating microbiota-derived metabolite signaling into such investigations will further facilitate the translational progression of GPs from preclinical exploration to clinical evaluation.

### 3.5 B lymphocytes

B cells mediate humoral immunity through antibody production (58). Given their pivotal role, dysregulated B-cell responses underpin diverse immunopathologies, including immunodeficiencies, autoimmunity, and allergic inflammation (26). Although research on GPs-mediated B-cell regulation lags behind studies on myeloid or T-cell subsets, emerging evidence suggests GPs potentiate humoral immunity. Shi et al. (70) demonstrated that GPs significantly augment the proliferation of LPS-stimulated murine splenic B cells in a dose-dependent manner (10–40 mg/mL), surpassing untreated controls. This indicates that GPs enhance B-cell activation under mitogenic challenge, likely contributing to robust humoral responses. Since B-cell activation is a precursor to antibody production, it is plausible that GPs-induced proliferation promotes the generation of antigen-specific plasma cells and immunoglobulins. However, direct evidence demonstrating that GPs enhance antibody affinity maturation, class switching, or long-lived plasma-cell differentiation remains lacking. Furthermore, it remains unclear if GPs directly activate B cells or require intermediary signals from T cells or dendritic cells. Resolving these questions via targeted B-cell assays and antibody profiling is crucial to define the contribution of GPs to adaptive immunity.

## 4 Gut microbiota-mediated immunity

The gut microbiota is integral to immune homeostasis, and its dysregulation underlies numerous diseases (79). Consequently, dysbiosis is implicated in a spectrum of immunopathologies, including IBD, allergies, autoimmunity, and carcinogenesis (13, 42, 94). The microbiota-related evidence for GPs-mediated immunomodulation should be interpreted according to experimental strength. Associations based solely on changes in microbial abundance should be distinguished from studies using antibiotic-mediated depletion, fecal microbiota transplantation (FMT), germ-free or gnotobiotic recipients, direct metabolite intervention, or defined microbial consortia. These approaches provide progressively stronger evidence for microbiota dependence and, where appropriately controlled, causal mediation. Nevertheless, each manipulation may also introduce confounding effects; therefore, causal claims should remain limited to the specific experimental model and endpoint examined. The strength of the available evidence differs substantially among studies. In a colon cancer model, Zhang et al. (115)reported that antibiotic-mediated microbiota depletion weakened the antitumor effect of GPs, whereas FMT restored this response. These findings support microbiota dependence in that model, although antibiotic depletion and FMT may have effects beyond selective removal or replacement of microbial communities. By contrast, Wu et al. (98) observed that dietary GPs improved growth performance and intestinal barrier function in laying hens together with increased abundance of commensal microbes. Because this study did not use microbiota depletion, FMT, germ-free recipients, metabolite supplementation or inhibition, or defined microbial consortia, the microbiota findings remain associative and cannot establish causal mediation. Song et al. (75) provided stronger mechanistic evidence in a cyclophosphamide-induced intestinal injury model. Low-molecular-weight GPs restored mucin secretion and altered the abundance of several taxa, including *Muribaculaceae*, *Alistipes*, *Lachnospiraceae*_*NK4A136*_group, *Ligilactobacillus*, and *Clostridia*_*vadinBB60*_group. Transfer of microbiota from GPs-treated donors into germ-free recipients reproduced selected immunostimulatory effects, supporting a transferable microbiota-dependent component in this murine model. Chen et al. (17) further associated GPs treatment with enrichment of *Dubosiella* and *Allobaculum* and increased butyrate and propionate concentrations. Unless these metabolites were directly administered, blocked, or otherwise manipulated, the SCFA data should be regarded as mechanistically suggestive rather than definitive evidence that SCFAs mediate the immune effects. Overall, current findings support a microbiota-associated or microbiota-dependent action of GPs in selected preclinical models, while evidence from defined-consortium or direct metabolite-intervention studies remains limited.

## 5 Pharmacokinetics and biological accessibility of orally administered GPs

For orally administered high MW GPs, pharmacokinetics and biological accessibility remain poorly characterized yet are central to interpreting their immunological effects. Because intact polysaccharides of several hundred kDa are generally not absorbed across the intestinal epithelium, the biological consequences of oral GPs administration should be dissected into sequentially linked compartments rather than inferred from plasma concentrations alone. The framework below draws on recent reviews of oral PK of medicinal polysaccharides (103, 106), while specific methods for resolving individual GPs fractions remain to be established. At the luminal level, intact GPs may exert local immunomodulation within the gut lumen by engaging PRRs on IECs or lamina propria immune cells. Although unabsorbed polysaccharides can produce local anti-inflammatory effects (106)), direct evidence of luminal PRR engagement by intact GPs is lacking; copurified low MW contaminants must therefore be excluded via fractionation and impurity controls. Beyond the lumen, high MW GPs translocation across the epithelium is constrained by the mucus layer, tight junctions, and lack of transporters. Identified absorption pathways include transcellular, paracellular, M cell, Peyer’s patch, and microbiota mediated routes (103). Fluorescent labeling demonstrates that pectin-type polysaccharides cross Caco-2 monolayers and accumulate in liver and kidney (109)); however, equivalent size specific data for GPs are absent, rendering any proposed barrier modulation effects a mechanistic hypothesis pending direct uptake assays.

Within the lower gastrointestinal tract, high MW GPs likely escape upper-GI digestion and reach the colon, where microbiota degrade them into oligosaccharides and SCFAs. Pectin-rich polysaccharides resist upper GI hydrolysis and yield substantial SCFAs *in vitro* (95). GalA-rich GPs pectic domains, such as those in GPS-1, are candidates for Bacteroides mediated utilization, but this requires experimental validation through antibiotic depletion, FMT, or defined consortium studies. Systemic exposure, if any, likely depends on absorption of these degradation products rather than the intact polymer. Low MW fractions and oligosaccharides may cross via paracellular or transcytotic routes, whereas native high MW chain absorption is limited (103). Dual labeling studies of glucans set a methodological precedent for probing absorption (46), but direct GPs evidence, including plasma and tissue concentration profiles and portal vein sampling, is essentially absent. Conventional PK metrics should not be extrapolated without validated detection of absorbed species.

Systemic immune outcomes may therefore arise via three principal routes. These include mucosal immune activation relayed lymphatically without systemic polymer exposure, SCFA and metabolite circulation, and genuine absorption of low MW fragments. Orally administered polysaccharides can reprogram gut microbiota and immunity to produce systemic effects (106), with Peyer’s-patch M-cell uptake providing a lymphatic conduit (47). Whether these routes operate for GPs remains unknown. Collectively, current evidence supports a compartmentalized model wherein high MW GPs act predominantly within the lumen and at the interface between the epithelium and the microbiota, with systemic effects relayed by microbial metabolites or limited low MW fragments. Definitive resolution requires coupled PK and immunology studies using labeled GPs of defined size fractions, portal and peripheral sampling, and germ-free models. Until such data emerge, each compartment’s contribution should be framed as a mechanistic hypothesis.

## 6 Applications

### 6.1 Therapeutic potential and translational gap of *Glycyrrhiza* polysaccharides

Licorice (*Glycyrrhiza uralensis*) is a premier medicinal herb used globally to treat inflammatory and hepatic disorders (104). Its therapeutic relevance extends globally; licorice root has been a staple in ancient Egyptian, Greek (Hippocratic), and early Chinese pharmacopeias (*Shennong Ben Cao Jing*) for millennia (9, 27). Traditionally indicated for inflammatory, respiratory, and gastrointestinal ailments, modern pharmacology attributes these effects to its anti-inflammatory, detoxifying, and analgesic properties (4, 67). The clinical evidence discussed in this section is restricted to GPs, namely carbohydrate-rich fractions that have been isolated from *Glycyrrhiza* species and evaluated as such. GPs should not be conflated with glycyrrhizin/glycyrrhizic acid, glycyrrhetinic acid, flavonoids, crude licorice extracts, or multi-component licorice preparations. Although glycyrrhizin-containing products have been investigated clinically, including in liver disease, such findings reflect the pharmacology of triterpenoid saponins or mixed preparations and cannot be used to infer the efficacy, mechanism, dose, or safety of GPs.

At present, the translational evidence for GPs is almost exclusively preclinical, and critical evaluation reveals substantial gaps. Defined GPs fractions have shown immunomodulatory, barrier-protective, antioxidant, and microbiota-related effects in cellular and animal studies; however, these findings have not been confirmed in adequately designed human trials. Moreover, many preclinical studies report general immunostimulatory endpoints rather than disease-specific or antigen-specific efficacy, and inconsistencies in GPs preparation, dose, and model make cross-study comparison difficult. The patent activity summarized in **Table 3** indicates technological and commercial interest but does not constitute clinical evidence of efficacy or safety. Therefore, GPs should currently be regarded as candidate immunomodulatory macromolecules rather than clinically established therapeutics, and their translational potential must be interpreted with appropriate caution.

**Table 3:** Patents related to *Glycyrrhiza* polysaccharide products.

No	Application	Main composition	Pharmacological properties	Publish number
1	Pharmaceutical	*Glycyrrhiza* polysaccharide	Preventing and/or treating liver injury	CN120570909A
2	Pharmaceutical	*Glycyrrhiza* polysaccharide	acute lung injury	CN120137069A
3	Skincare products	*Dendrobium officinale* polysaccharides, *peach gum* polysaccharides, *Polygonatum sibiricum* polysaccharides, and licorice polysaccharides	anti-aging	CN119033625A
4	Food	*Glycyrrhiza* polys	antioxidant	CN117752029A
5	Food	Sticky corn, licorice polysaccharide, alkaline water pH, calcium, islet fruit, salt	immunomodulation	CN116918970A
6	Health product	*Ganoderma lucidum* polysaccharides, ginseng, *Astragalus* polysaccharides, and licorice polysaccharides	anti-fatigue	CN116672374A
7	Animal Science	Lotus leaves, cypress leaves, licorice polysaccharides, vitamins	immunomodulation	CN116350680A
8	Biological product	Serum-free basal medium, *Astragalus* membranaceus flavone, *Astragalus* polysaccharide, *Glycyrrhiza* polysaccharide, *Salvia miltiorrhiza* polysaccharide, *Ganoderma lucidum* polysaccharide	immunomodulation	CN114517181A

Patent filings indicate proposed uses or formulations and should not be interpreted as clinical evidence. For multi-component products, the listed biological activity cannot be attributed specifically to *Glycyrrhiza* polysaccharides unless the patent provides component-resolved experimental evidence.

### 6.2 Vaccine adjuvant

Vaccines represent the most cost-effective public health intervention against infectious diseases, with adjuvants serving as critical immunoenhancing components (30). Defined as immunobiologic agents that potentiate antigen-specific adaptive immunity, adjuvants address the inherent limitations of subunit vaccines (44). Plant polysaccharides, including GPs, have been investigated as potential immunomodulatory components for vaccine formulations (52). However, their suitability as vaccine adjuvants must be evaluated separately from their general immunostimulatory activity, and current evidence for GPs remains predominantly preclinical. Notably, isolated immunostimulatory effects are insufficient to validate a candidate agent as a functional vaccine adjuvant. Qualified adjuvants must satisfy multiple rigorous criteria, including the capacity to amplify antigen-specific humoral and cellular immune responses, sustain long-term immunological memory, exhibit favorable safety with negligible local and systemic reactogenicity, facilitate antigen dose sparing, and maintain compatibility with various antigen formulations and delivery routes. Most GPs studies have demonstrated broad immunostimulatory effects, such as cytokine induction, macrophage activation, dendritic-cell maturation, or enhanced lymphocyte responses. These findings should not be equated with definitive vaccine-adjuvant activity. Evidence supporting antigen-specific enhancement, durable immunological memory, dose sparing, reduced reactogenicity, formulation stability, and superiority or non-inferiority to established adjuvants remains limited. Although accumulating preclinical findings from murine and poultry models have demonstrated the immunomodulatory capacity and promising adjuvant potential of GPs, such beneficial effects have not yet been validated in clinical trials. In poultry, Zhou et al. (122) reported that co-administration of the purified GPs fraction GPS-1 with Newcastle disease virus (NDV) vaccine improved growth performance and increased NDV-specific antibody and cytokine responses. These findings support the veterinary immunomodulatory and adjuvant potential of GPS-1 in chickens, but they should not be extrapolated directly to mammalian or human vaccine development. Building upon these observations, Wu et al. (96) reported that oral GPS-1 at 300–600 mg/kg enhanced antibody and cellular immune responses to NDV vaccination in chickens. Because the study used a relatively high oral dose, the translational relevance of this dosing regimen remains uncertain. Further studies should establish oral bioavailability, dose–response relationships, pharmacokinetic or exposure parameters, batch-to-batch reproducibility, and compatibility with standard vaccination protocols. GPS-1 boosted hemagglutination inhibition titers and NDV-specific IgG/IgA levels. Crucially, it expanded splenic CD4^+^ and CD8^+^ T-cell subsets and elevated IL-2, IL-4, IL-17, and IFN-γ, suggesting enhancement of both humoral and cellular immune responses in this poultry model. Despite these encouraging results, several key limitations restrict the current understanding of GPs adjuvant properties. First, both relevant studies were established based on poultry Newcastle disease vaccine models, rather than mammalian or human experimental systems. Second, these studies only evaluated NDV-specific antibody titers and cytokine levels as primary outcomes, lacking rigorous assessment of long-term immunological memory. Third, no head-to-head comparisons with clinically licensed veterinary or human adjuvants have been performed. Fourth, the oral administration strategy adopted in Wu et al. still requires systematic validation regarding its bioavailability and experimental reproducibility (96).

A distinct group of studies has incorporated GPs into nanoparticle or liposomal delivery systems. These investigations should be considered nano-vaccine or antigen-delivery studies rather than direct evidence for the performance of unformulated GPs as conventional vaccine adjuvants, because the observed effects may depend on the combined properties of the GPs, antigen, carrier, particle size, surface characteristics, and release profile. Yao et al. (105) identified NGUP, a unique GPs, which activates DCs via the TLR4/MyD88/TRAF6/NF-κB axis. To enhance targeting, NGUP was formulated into a lymph-node-homing liposomal nanovaccine (NGUPL@OVA). In the reported mouse study, NGUPL@OVA increased dendritic-cell maturation relative to empty liposomes and alum-containing controls and enhanced antigen-specific CD8^+^ T-cell and central-memory T-cell responses. The formulation also showed strong prophylactic and therapeutic antitumor effects in melanoma-bearing mice. Nevertheless, these results represent a comparison within a murine tumor-vaccine model and do not establish superiority over alum in infectious-disease vaccination. The contribution of NGUP, the liposomal carrier, and OVA antigen delivery should be resolved using appropriate component-matched controls. Beyond liposomes, Zhao et al. (125) engineered GUPS-AuNPs, conjugating GPs to gold nanoparticles to create a bifunctional platform integrating adjuvant signaling with antigen delivery. *In vitro*, the GPs–gold nanoparticle formulation increased dendritic-cell activation markers and inflammatory cytokine production and promoted T-cell priming in the reported experimental system. However, these effects cannot be attributed exclusively to GPs without comparisons with unconjugated GPs, gold nanoparticles alone, antigen alone, and other component-matched formulations. *In vivo*, a DC-based vaccine using GUPS-AuNPs (GUPS-AuNP+E7pro-DC) significantly suppressed TC-1 tumor growth. This correlated with increased splenic CD4^+^/CD8^+^ T-cell activation, reduced Tregs, and robust IFN-γ-secreting CD8^+^ T-cell responses. This approach transforms GPs from simple PAMP-like stimulants into sophisticated delivery systems that synchronize antigen cross-presentation and APC licensing. While these nanoparticle-based GPs studies provide innovative insights, their findings require prudent scientific interpretation due to several inherent limitations. First, these investigations adopt therapeutic cancer vaccine models, rather than prophylactic vaccines against infectious diseases. Second, the observed antitumor efficacy stems from the synergistic effects of GPs, nanoparticle vectors, and tumor antigens, making it difficult to independently attribute adjuvant functions specifically to GPs components. Third, all relevant experiments have been limited to murine models, with no valid evidence from large animal or human studies. Finally, comprehensive safety and toxicological assessments are still absent, including evaluations of local injection-site reactogenicity and systemic adverse reactions.

In summary, GPs show preliminary preclinical potential in two distinct settings: conventional or orally administered GPs preparations evaluated with veterinary vaccines, and GPs-containing nanoparticle formulations evaluated mainly in murine cancer-vaccine models. These evidence streams should not be combined because they differ substantially in species, antigen, route of administration, formulation, endpoint, and disease model. Nevertheless, current supporting evidence remains preliminary. Most existing studies only validate generalized immunostimulatory effects rather than robust antigen-specific adjuvant activity, while data regarding long-term immunological memory are scarce and clinical trials in human subjects remain absent. Future studies should proceed through a clearly separated evidence hierarchy. First, veterinary studies should evaluate antigen-specific protection, dose–response relationships, and comparison with licensed veterinary adjuvants. Second, mouse studies should distinguish conventional GPs preparations from GPs-containing nanovaccines and should include component-matched controls. Third, studies in larger animals should assess immunogenicity, protection, dose sparing, reactogenicity, pharmacokinetics, and formulation stability. Only after adequate toxicology, manufacturing consistency, and reproducible comparative efficacy have been established should human adjuvant-development studies be considered. At present, no clinical evidence demonstrates the safety or efficacy of GPs as human vaccine adjuvants.

### 6.3 In the field of agriculture

Escalating global scrutiny of food safety standards, animal welfare ethics, and the ecological threat of antimicrobial resistance has catalyzed an urgent search for sustainable, non-antibiotic growth promoters within livestock production and aquaculture systems (86). Within this context, GPs have emerged as promising phytogenic feed additives with potential to reduce reliance on conventional antibiotics, based on preclinical evidence from livestock and poultry studies. Converging preclinical evidence from animal production studies indicates that dietary GPs supplementation improves zootechnical performance across diverse livestock and poultry systems (91, 119). In quail, dietary *Glycyrrhiza* polysaccharide (GCP) at 500–1500 mg/kg significantly enhanced female growth performance, increasing final body weight to 246.52 g while reducing cumulative mortality to 4.53% during the 1–50 day rearing period (111). These effects coincide with prebiotic-like changes, including reshaping of the gut microbiota, increased SCFA concentrations, and enhanced intestinal barrier function. However, because microbiota depletion, FMT, germ-free transfer, direct metabolite intervention, and defined-consortium experiments were not performed, these findings demonstrate association rather than causal mediation. Beyond improvements in growth performance and productivity, accumulating evidence suggests that the beneficial effects of GPs are closely associated with their capacity to regulate intestinal health. Consistent with these observations, Li et al. (43) identified 1000 mg/kg as the optimal GCP inclusion rate in post-weaning piglets, significantly enhancing average daily gain and reducing intestinal injury while priming innate immune defenses during this vulnerable window. Similarly, in a complementary avian study, Wu et al. (98) demonstrated that GPS supplementation boosts intestinal mucosal immunity in broilers, accompanied by increased secretory IgA levels, enriched butyrate-producing Butyricicoccus, and balanced expression of immune-related cytokines (IL-2, IL-4, and IFN-γ). Notably, these observations remain correlational, as the study did not perform microbiota manipulation assays to verify whether microbial remodeling or altered short-chain fatty acid production directly drives mucosal immune enhancement. These immunological alterations were accompanied by an increased CD4^+^/CD8^+^ T-cell ratio. However, the absence of microbiota-manipulation experiments prevents attribution of these immune effects specifically to microbial remodeling or SCFA production, and the findings should therefore be interpreted as preliminary evidence of a microbiota-associated immunomodulatory effect, rather than as evidence that GPs can replace therapeutic antibiotics or antibiotic growth promoters. GPs may have potential to reduce reliance on antibiotic growth promoters, but this possibility requires confirmation in well-controlled infection-challenge studies and direct comparative trials. Furthermore, GPs effectively preserve post-slaughter meat quality. Their potent free-radical scavenging capacity suppresses myofibrillar lipid oxidation, thereby sustaining meat color stability, tenderness, and water-holding capacity to prolong shelf life (63). Nevertheless, several key limitations restrict the translational and commercial applicability of current agricultural evidence. First, most livestock studies are performed under customized farming conditions with inconsistently prepared GPs samples, hindering cross-study comparison and universal result generalization. Second, the molecular mechanisms responsible for GPs-mediated production improvements remain poorly elucidated. Third, systematic evaluations regarding long-term animal health outcomes, animal product safety, and environmental influences are still absent. Fourth, standardized dosing regimens, optimized formulations, and unified quality control protocols for commercial GPs application have not yet been established. Collectively, existing preclinical and production-animal studies suggest that GPs may improve selected indicators of animal productivity, intestinal health, immune status, and meat quality. However, these findings do not establish that GPs can directly replace antibiotics or antibiotic growth promoters. Rather, GPs may represent a potential nutritional strategy for reducing reliance on antibiotic growth promoters, provided that their efficacy, safety, consistency, and effects on antimicrobial use are validated in standardized, adequately powered, and well-controlled studies, including infection-challenge models and direct comparator trials.

## 7 Safety and toxicity concerns

Safety considerations should be evaluated in a constituent-specific manner rather than generalized across *Glycyrrhiza*-derived preparations. Most well-recognized adverse effects of licorice, including pseudo-hyperaldosteronism, hypertension, hypokalemia, and interactions involving drug-metabolizing enzymes or transporters, have been primarily attributed to glycyrrhizin/glycyrrhizic acid and its metabolite 18β-glycyrrhetinic acid rather than to polysaccharide fractions (14, 15, 53, 60). Mechanistically, glycyrrhetinic acid-mediated inhibition of 11β-hydroxysteroid dehydrogenase type 2 results in excessive activation of mineralocorticoid signaling, providing a major explanation for glycyrrhizin-associated adverse effects (28, 84). These findings are highly relevant for the safety evaluation of glycyrrhizin-containing licorice products; however, they should not be interpreted as direct evidence of toxicity associated with purified GPs.

In contrast, the safety profile of structurally defined GPs remains poorly characterized. While current *in vitro* and animal studies support their acceptable tolerability at tested doses, systematic assessments regarding long-term toxicity, pharmacokinetics, immunotoxicity, and clinical safety are still lacking. GPs isolates exhibit pronounced heterogeneity in molecular features, compositional profiles, purification strategies, and residual contaminants, including low-molecular-weight metabolites, proteins, glycoproteins, and endotoxin. Such variability substantially alters GPs immunological behaviors. Given that endotoxin strongly triggers TLR4 signaling and residual impurities independently regulate immune responses, rigorous purity validation is critical to confirm the polysaccharide-specific immunological functions of GPs. Future GPs safety evaluations therefore require comprehensive chemical characterization, impurity identification, component-based toxicological analysis, pharmacokinetic investigation, and standardized clinical monitoring. Furthermore, safety and dose thresholds defined for glycyrrhizin-related products merely provide background references and cannot be directly adopted for licorice-derived GPs.

## 8 Future perspectives and challenges

Licorice-derived GPs represent promising immunomodulatory macromolecules that regulate immune responses via direct cellular signaling and indirect remodeling of the microbiota–gut–immune axis. Nevertheless, multiple fundamental and translational challenges hinder their clinical application. First, the inherent structural heterogeneity and prominent batch-to-batch variability of natural GPs impair bioactivity consistency and impede unified quality control, constituting a major translational bottleneck. Second, current SARs analyses only reveal correlational associations, without definitive causal evidence. The overlapping structural features of high-molecular-weight and highly branched GPs fractions make it difficult to distinguish the independent and synergistic contributions of molecular weight and structural topology to bioactivity. Third, the *in vivo* physicochemical behaviors of orally administered GPs remain poorly defined, with critical knowledge gaps in gastrointestinal biotransformation, glycan fragment bioavailability, and dynamic GPs–microbiome interactions. Fourth, the lack of rigorous randomized controlled trials leaves human efficacy unvalidated, substantially limiting clinical progression. Fifth, standardized purity-control and effect-attribution protocols are currently absent in GPs research. Residual endotoxin, proteins, glycoproteins, and small-molecule metabolites can independently activate pattern recognition receptor (PRR)-mediated inflammatory pathways, confounding the immunological outcomes of crude GPs preparations. Future studies must routinely adopt systematic controls, including LAL endotoxin quantification, polymyxin B neutralization, proteinase K digestion, extensive dialysis, and structure–activity validation, to confirm polysaccharide-specific functions. Establishing such standardized protocols is indispensable for reliable mechanistic interpretation and clinical translation.

Addressing these limitations requires interdisciplinary strategies integrating glycobiology, systems biology, and clinical research. (1) Biosynthetic profiling: Multi-omics approaches can decipher the genetic and enzymatic regulatory networks underlying GPs biosynthesis, including glycosyltransferase profiling, enzymatic modification validation, and nucleotide-sugar donor tracing (49), to support precise GPs biosynthesis. (2) Homogeneous GPs standardization: Controlled enzymatic hydrolysis and high-resolution fractionation enable the generation of chemically defined, homogeneous GPs oligomers. Such standardized fractions facilitate high-precision SARs characterization and allow the design of simplified glycan mimetics based on core active pharmacophores, promoting the development of next-generation glycan-based immunomodulators. (3) Structural conformational analysis: Integrative structural characterization using circular dichroism, NMR spectroscopy, and molecular dynamics simulations helps monitor GPs conformational dynamics under physiological conditions. Combined with surface plasmon resonance and saturation transfer difference NMR, these techniques enable quantitative evaluation of GPs–PRR binding thermodynamics. (4) Microbiome–immunomodulation association: As prebiotic regulators, GPs exert strain-specific fermentability dependent on their structural epitopes. Clarifying the linkage between GPs-mediated short-chain fatty acid production and host immune regulation will build predictive mechanistic models for translational research. Overcoming these challenges will be necessary to advance GPs from traditional extracts toward candidate precision biologics for preventive medicine and immunotherapy, a transition that remains dependent on future clinical validation.

## 9 Conclusion

Importantly, the immunomodulatory evidence discussed in this review is specifically based on structurally characterized GPs preparations and should be interpreted independently from the pharmacological and clinical literature on other *Glycyrrhiza*-derived constituents, including glycyrrhizin, glycyrrhetinic acid, flavonoids, crude extracts, and multi-component formulations. Although these non-polysaccharide licorice products have demonstrated therapeutic effects in certain clinical settings, such evidence cannot be directly attributed to GPs or used to establish their clinical efficacy, mechanism of action, or safety profile. Furthermore, because many of the cited immunological studies were conducted with variably purified GPs preparations, the possibility that residual endotoxin, proteins, glycoproteins, or low-molecular-weight compounds contribute to the reported effects must be acknowledged; rigorous purity controls and effect-attribution experiments are essential to establish that the immunomodulatory activity is specifically attributable to the polysaccharide fraction.

GPs constitute a valuable class of plant-derived immunomodulatory macromolecules with broad biological activity and promising therapeutic potential, although the supporting evidence remains predominantly preclinical. Extensive studies have revealed that their immune-regulating activities arise from the interplay between intrinsic structural complexity and biological recognition processes. Molecular characteristics, including MW, monosaccharide composition, glycosidic linkage patterns, branching topology, and supramolecular conformation, are associated with differences in receptor-pathway readouts, immune-cell responses, and microbiota-related outcomes. Their individual and combined causal contributions remain to be established using structurally controlled GPs preparations and direct target-engagement experiments.

Nevertheless, the clinical translation of GPs remains limited by structural heterogeneity, insufficient quality-control strategies, incomplete understanding of precise structure–activity relationships, and the scarcity of human clinical evidence. Future investigations should integrate high-resolution structural analysis, multi-omics technologies, computational modeling, and clinical studies to establish predictive relationships between glycan structures and immune functions. Such advances will enable the rational design of standardized and mechanism-driven GPs-based candidate immunomodulators, thereby promoting their progression from preclinical evidence toward clinical evaluation as potential next-generation natural immune therapeutics.
